# Oral administration of *Jumihaidokuto* inhibits UVB-induced skin damage and prostaglandin E2 production in HR-1 hairless mice

**DOI:** 10.1007/s11418-020-01465-y

**Published:** 2020-11-17

**Authors:** Kenta Murata, Manami Oyama, Misaki Ogata, Nina Fujita, Ryuji Takahashi

**Affiliations:** Kampo Research Laboratories, Kracie Pharma, Ltd., 3-1 Kanebo-machi, Takaoka, Toyama 933-0856 Japan

**Keywords:** *Jumihaidokuto*, UVB, Collagen, Elastin, Prostaglandin E2

## Abstract

This study was conducted to investigate whether and how *Jumihaidokuto* (JHT), a traditional Chinese medicine, prevents UVB-induced skin damage in male HR-1 hairless mice. JHT has been traditionally prescribed for patients presenting skin disorders with redness and swelling, and, in Japan, it is approved for prescription to patients with acute and/or purulent skin disorders, hives, acute eczema, and athlete’s foot. Considering the traditional use of JHT, we hypothesized that oral administration of JHT might emerge as an effective strategy to prevent UVB-induced skin damage, such as edema and erythema. Here, we pretreated mice with JHT (1000 mg/kg, p.o.) for 3 weeks and then administered a single dose of UVB irradiation (250 mJ/cm^2^) on the dorsal skin. UVB irradiation increased the erythema index and transepidermal water loss (TEWL) and decreased the skin water content in the epidermis at 72 h post-irradiation. JHT treatment inhibited the increase of TEWL and the loss of water content in the epidermis, but not the elevation of the erythema index. Moreover, administration of JHT suppressed UVB-induced epidermal hyperplasia by blocking the proliferation of keratinocytes and also inhibited irradiation-triggered reduction of collagen fibers and infiltration of immune cells into the dermis. Lastly, administration of JHT suppressed UVB-induced production of proinflammatory mediators, such as prostaglandin E2 and interleukin-1β. These results suggest that JHT prevents UVB-induced skin damage and that the underlying mechanism involves the inhibition of proinflammatory mediators.

## Introduction

The ultraviolet (UV) light that reaches the earth’s surface is composed of 90% UVA (320–400 nm) and 10% UVB (280–320 nm). Acute exposure of the skin to UVB produces various harmful effects, including inflammation, impaired skin barrier function, photoaging, and carcinogenesis. Skin inflammation is characterized by erythema, swelling, edema, and itching. In UVB-induced skin inflammation, oxidative stress plays a pivotal role in initiating and driving the cellular response to UVB irradiation [1] and enhances the production of diverse proinflammatory mediators, such as prostaglandin (PG) E2 [2], a key signaling molecule in UVB-induced inflammation. PGE2, the most abundant PG in humans, is generated by the action of cyclooxygenase (COX) enzymes and plays a crucial role in initiating various aspects of the inflammatory response. For example, PGE2 increases vascular permeability in blood vessels to facilitate the infiltration of neutrophils and macrophages from the bloodstream, and this leads to swelling and edema at the site of inflammation after UVB irradiation [3, 4]. Moreover, photoaged skin is histologically characterized by the degradation of extracellular matrix (ECM), such as the loss of dermal collagen and the accumulation of abnormal elastic fibers (termed “solar elastosis”). Inflammation and immune-cell infiltration activate various matrix metalloproteinases (MMPs); this leads to aberrant degradation of ECM and accumulation of nonfunctional matrix components in the skin [5, 6]. MMPs are a family of zinc-dependent endopeptidases that are critically involved in the degradation of ECM. Furthermore, UVB-induced accumulation of elastic fibers is considered to occur because of the overproduction and/or decreased degradation of elastic fibers; accordingly, the expression of elastin and fibrillin mRNAs is reported to be increased in photodamaged skin [7], and the major source of skin elastase is reported to be the neutrophils and mast cells that infiltrate the dermal layer following UVB exposure [5, 8, 9].

Plant extracts and herbs containing numerous types of compounds have long been administered for treating different diseases. Since oxidative stress plays a central role in UVB-induced inflammation, the use of botanical products that exhibit strong antioxidant activity has attracted considerable research interest in efforts to reduce the risk of skin disorders induced by UVB irradiation [10]. Topical application of natural compounds or extracts that produce protective effects against UVB-induced inflammation has been widely reported, and myriad sun creams containing such compounds and extracts have been developed [2, 11, 12]. Recently, certain plant extracts, such as *Polypodium leucotomos* extract, were reported to ameliorate UVB-induced skin inflammation and carcinogenesis when administered orally [13]. For this reason, several plant extracts have also been developed as “drinkable sunscreens.”

*Jumihaidokuto* (JHT), a traditional Chinese medicine composed of 10 dried medical herbs, has been traditionally prescribed for patients presenting skin disorders with redness and swelling. In Japan, JHT is approved for prescription to patients with ailments, such as acute and/or purulent skin disorders, hives, acute eczema, and athlete’s foot. Furthermore, JHT has also been reported to be useful for the clinical treatment of palmoplantar pustulosis [14], chronic eczema [15], and acne vulgaris [16]. In an in vivo study, JHT suppressed *Propionibacterium acnes*-induced dermatitis by modulating the functions of macrophages [17], and in in vitro studies, JHT was found to enhance neutrophil functions, such as chemotaxis and phagocytosis [18], and to inhibit the induction of neutrophil-derived and cell-free reactive oxygen species (ROS) in a dose-dependent manner [19]. Considering the traditional use of JHT, we hypothesized that oral administration of JHT could be developed into an effective strategy for preventing UVB-induced skin damage.

We conducted this study to clarify whether and how JHT treatment mitigates UVB-induced skin inflammation in hairless mice.

## Materials and methods

### Animals

Six-week-old male hairless HR-1 mice, weighing 19.5–23.1 g (mean ± standard deviation 21.5 ± 1.2 g), were purchased from SLC (Shizuoka, Japan), housed in sterilized polypropylene cages (4–5 mice/cage) at 24 ± 2 °C under a 12/12-h light/dark cycle (lights on from 08:00 to 20:00 h), and provided laboratory pellet chow (CE-2, Clea Japan Inc., Tokyo, Japan) and water ad libitum. Before starting the experimental procedures, the mice were acclimatized to the room for 1 week. All efforts were made to minimize suffering to the animals and the number of animals used. This study was conducted in accordance with the principles of the Basel Declaration and recommendations of guidelines for Proper Conduct of Animal Experiments, the Experimental Animal Care Committee of Kracie Pharma, Ltd. (Toyama, Japan). The protocol was approved by the Experimental Animal Care Committee of Kracie Pharma Ltd.

### Plant materials and extract preparation

JHT is composed of 10 dried medical herbs—*bupleurum root*, *platycodon root*, *cnidium rhizome*, *poria sclerotium*, *saposhnikovia root*, *ginger*, *schizonepeta spike*, *aralia rhizome*, *cherry bark*, and *glycyrrhiza* (Table 1)—and is supplied by Kracie Pharma Ltd., as a formulation (EK-6), which contains 3900-mg JHT extract in 6000-mg formulation. The formulation also contains magnesium stearate, cellulose, lactose hydrate, and hydrated silicon dioxide as additives. Each plant material was identified based on its external morphology and was authenticated based on compound markers of plant specimens according to the method of Japanese Pharmacopoeia and our company’s standard. EK-6 (Lot No. 11J72) was suspended in distilled water immediately before use and was administered once a day at a dose of 1000 mg/kg bodyweight/day through gavage. The dose was determined with reference to the formula dB = dA × KB/KA, where dB is the daily dose, in milligram per kilogram of mouse bodyweight; dA is the daily dose, in milligram per kilogram of adult human bodyweight; and the final term is a constant, with KB = 1.0 and KA = 0.11. For calculation, the following values were used: mouse weight, 0.03 kg; normal EK-6 dosage for an adult human, 6000 mg/day; and adult human weight, 60 kg; when converted into dosage for mice, these values yield a dose of 909 mg/kg mouse bodyweight/day.

**Table 1:** Medical herb composition of *Jumihaidokuto* (JHT)

Common name	Weight (g)
Bupleurum root	2.5
Platycodon root	2.5
Cnidium rhizome	2.5
Poria sclerotium	2.5
Saposhnikovia root	2.5
Glycyrrhiza	1.5
Ginger	1.0
Schizonepeta spike	1.5
Aralia rhizome	1.5
Cherry bark	2.5

### High-performance liquid chromatography (HPLC) analysis of JHT

JHT extract was mixed and shaken with 50% MeOH and centrifuged. The supernatant was subjected to high-performance liquid chromatography (HPLC). The HPLC profile of YKSCH was obtained using a Shimadzu LC-30AD liquid chromatography equipped with a SPD-M30A detector over a scan range of 200–700 nm using a reversed-phase column (YMC-pack ProC18, 2.0 mm i.d. × 150 mm, 12 nm, column temperature 20 °C). The solvent system consisted of solvent A (0.1% formic acid in acetonitrile) and solvent B (0.1% formic solution) and was used at a flow rate of 0.2 mL/min; the ratio of solvent A was increased from 5 to 70% over 90 min.

### UVB irradiation and measurement of skin moisture content in dermis and epidermis, transepidermal water loss (TEWL), and erythema index

Mice were irradiated using a handheld UVB lamp, UVM-57, under isoflurane anesthesia; a single dose of UVB (250 mJ/cm^2^) was administered after 3 weeks of JHT treatment. Before and after UVB irradiation, the following skin parameters were measured under isoflurane anesthesia (all detections were performed thrice on the dorsal skin of each hairless mouse, using appropriate devices): skin moisture in the epidermis, using a corneometer, CM825 (Courage and Khazaka Electronics, Cologne, Germany); skin moisture in the dermis, using a moisturemeter-D (Delfin Technologies, Kuopio, Finland); TEWL, using a TM300 tewameter (Courage and Khazaka Electronics); and erythema index, using a mexameter, MX18 (Courage and Khazaka Electronics). After the measurements, mice were killed under isoflurane anesthesia and their dorsal skin was collected. Changes in skin moisture and erythema index were both calculated using the following formula: (index change) = (value at 72 h after UVB irradiation) − (value before UVB irradiation).

### Histological analysis

Mice were killed under isoflurane anesthesia, and the dorsal skin was collected and immediately immersed in Bouin’s fluid for 24 h at 4 °C. Subsequently, paraffin-embedded tissue sections (5-μm thick) were stained with hematoxylin and eosin (HE) and Masson’s trichrome to evaluate skin thickness and collagen fibers, respectively. Epidermal thickness was calculated by dividing the area of the epidermis by the length of the basal layer, and dermal thickness was calculated by dividing the area of the dermis by the length of muscle. Collagen density was calculated using Fiji, an open-source platform for biological-image analysis [20].

### Immunohistochemical analysis

Paraffin-embedded tissue sections fixed in Bouin’s fluid were deparaffinized and hydrated. For the analysis of Ki67, endogenous peroxidases were inhibited by incubating the sections for 30 min with freshly prepared 0.3% hydrogen peroxide (H_2_O_2_) in methanol, after which the sections were treated with 0.1% trypsin at 37 °C for 30 min. Nonspecific staining was blocked with 5% goat serum for 60 min at room temperature, and then the sections were sequentially incubated overnight with rabbit polyclonal anti-Ki67 antibody (1:1000; Abcam, Cambridge, UK) and with horseradish peroxidase (HRP)-conjugated anti-rabbit secondary antibody (Agilent, Santa Clara, CA, USA) at room temperature for 1 h.

For the staining of Ly-6C/-6G and Iba-1, sections were treated with citrate buffer overnight at 60 °C after inhibition of endogenous peroxidases, incubated in blocking solution to eliminate nonspecific staining, and then with rat monoclonal anti-Ly-6C/-6G antibody (1:400; Abcam) or rabbit polyclonal anti-Iba-1 antibody (1:2000; Abcam) overnight at 4 °C. Finally, the sections were incubated at room temperature for 1 h with HRP-conjugated anti-rabbit secondary antibody or anti-rat secondary antibody (MBL Co. Ltd., Aichi, Japan).

For 8OH-DG and CPD immunostaining, a “mouse on mouse” (M.O.M.) staining kit (Vector Laboratories Inc., Burlingame, CA, USA) was used as per the product manual: tissues sections were treated with 0.1% trypsin for 30 min at 37 °C, blocked with the M.O.M. kit blocking reagent for 1 h at room temperature to eliminate nonspecific staining, and then incubated with mouse anti-8OH-DG antibody (1:500; Nikken Seil Co. Ltd., Tokyo, Japan) or mouse anti-CPD antibody (1:1000; Cosmo Bio Co. Ltd., Tokyo, Japan) in M.O.M. antibody diluent reagent for 1 h at room temperature. Endogenous peroxidases were inhibited by incubating sections with 0.3% H_2_O_2_ in methanol for 30 min, and the sections were then incubated for 30 min with HRP-conjugated anti-mouse secondary antibody contained in the M.O.M. kit or Alexa fluor 488 anti-mouse secondary antibody (Thermo Fisher Scientific, Waltham, MA, USA).

Color development for all immunostaining was performed using diaminobenzidine substrate (Sigma-Aldrich, St. Louis, MO, USA). The densities of cells positive for Ki67, 8OH-DG, and CPD were calculated by dividing the counted number of cells by the basal layer length of the counted area, whereas the densities of cells positive for Ly-6C/-6G and Iba-1 were calculated by dividing the counted number of cells by the volume of the counted area.

### Western blot analysis

Frozen mouse dorsal skin samples were homogenized (at 10 mL/g) in RIPA buffer (Fujifilm, Tokyo, Japan) supplemented with a protease-inhibitor cocktail (Nacalai Tesque, Kyoto, Japan) and phosphatase-inhibitor cocktail (Nacalai Tesque). Lysates were centrifuged at 15,000 × *g* for 20 min at 4 °C, and then 10-μg aliquots of protein were separated on 10–20% SDS–polyacrylamide gels, transferred onto polyvinylidene difluoride membranes (Immobilon-P; Merck Millipore, Burlington, MA, USA), and immunoblotted with the following primary antibodies: rabbit anti-MMP-9 polyclonal antibody (1:1000; Gene Tex, Hsinchu, Taiwan), rabbit anti-COX-2 polyclonal antibody (1:1000; Cell Signaling Technology (CST), Danvers, MA, USA), rabbit anti-catalase polyclonal antibody (1:1000; Gene Tex), rabbit anti-SOD1 polyclonal antibody (1:1000; Gene Tex), rabbit anti-SOD2 polyclonal antibody (1:1000; Gene Tex), and mouse anti-β-actin monoclonal antibody (1:1000; CST). The secondary antibodies used were HRP-conjugated goat anti-rabbit IgG (1:5000; CST) or goat anti-mouse IgG (1:5000; CST). Immunoreactive bands were visualized using an Amersham Imager 680 (GE Healthcare, Chicago, IL, USA). Band intensity was measured using Fiji.

### Real-time PCR

Total RNA was isolated from frozen skin tissue samples using a Genelute mammalian total RNA miniprep kit (Sigma-Aldrich), and then mRNA was reverse-transcribed into cDNA using ReverTra Ace (TOYOBO, Osaka, Japan). Quantitative real-time PCR was performed using TB Green Premix Ex Taq II (Takara, Japan), employing the following amplification protocol: 40 cycles of 95 °C for 5 s and 60 °C for 30 s; a single fluorescence measurement was used. Melting curve analysis, for which the temperature was increased from 60 to 95 °C at a heating rate of 0.1 °C/s and continuous fluorescence measurement was done, revealed a single, narrow peak for the suspected fusion temperature. The following primers were used for real-time PCR: mouse IL-1β: forward, 5ʹ-TCCAGGATGAGGACATGAGCAC-3ʹ; reverse, 5ʹ-GAACGTCACACACCAGCAGGTTA-3ʹ; and mouse β-actin: forward, 5ʹ-ACCTTCTACAATGAGCTGCG-3ʹ; reverse, 5ʹ-CTGGATGGCTACGTACATGG-3ʹ.

### PGE2 measurement

Skin PGE2 level was measured by performing ELISA with an Enzyme Immunoassay Kit Correlate PGE2 (Assay Designs Inc., Ann Arbor, MI, USA), following the instructions in the product manual. Briefly, frozen tissue samples were homogenized with 10 μg/mL indomethacin, the lysates were centrifuged at 15,000 × *g* for 20 min at 4 °C, and the supernatants were mixed with 2 M HCl and allowed to sit for 15 min at 4 °C. After centrifugation, the supernatants were filtered using C18 reversed-phase extraction columns, the extracts were evaporated under a stream of nitrogen, and dried samples were mixed with the supplied assay buffer. These extracts were used as the samples.

### Elastin measurement

Elastin levels in mouse skin samples were measured using a Fastin elastin assay kit (Biocolor Ltd., Carrickfergus, UK), following the instructions in the product manual. Briefly, 10 mg samples of frozen tissue were homogenized in 1 mL of PBS and then mixed with 1.0 M oxalic acid and incubated at 100 °C for 60 min, and the lysates were centrifuged at 10,000 rpm for 10 min; the supernatants were collected and mixed with 0.25 M oxalic acid and centrifuged again. The final collected supernatants were used as the samples.

### Statistical analysis

All statistical analyses were performed using EZR (Saitama Medical Center, Jichi Medical University, Saitama, Japan), a graphical user interface for R (The R Foundation for Statistical Computing, Vienna, Austria). More precisely, EZR is a modified version of R commander designed to add statistical functions frequently used in biostatistics [21]. All data are expressed as means ± standard error of the mean (SEM). Statistical comparisons were performed using one-way analysis of variance followed by Student’s *t* test or Tukey test. Differences were considered significant at *p* < 0.05.

## Results

### HPLC analysis of JHT

**Fig. 1:**
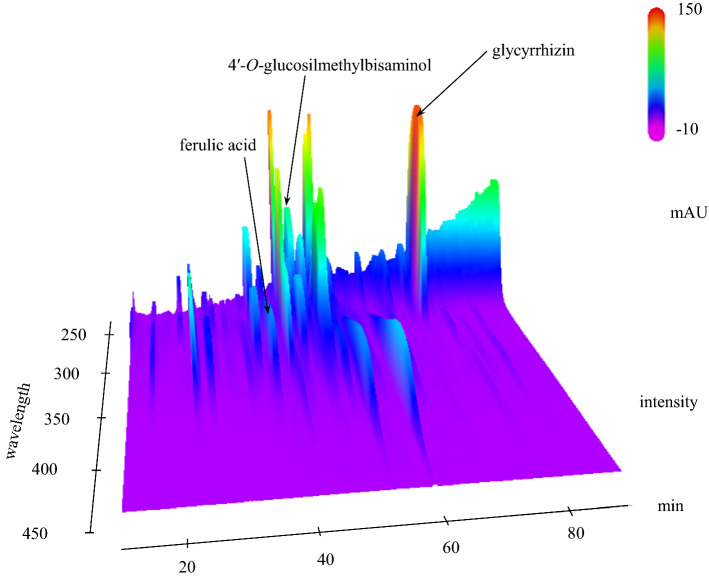
3D-HPLC profile of *Jumihaidokuto* (JHT). Chemical markers (ferulic acid, glycyrrhizic acid, 4′-o-glucosyl-5-methylvisamminol) in HPLC profiles were identified based on comparison with the retention times and UV spectra (220–400 nm) of their reference standards

Figure 1 shows a 3D-HPLC profile of JHT together with its chemical analysis. Chemical markers, such as ferulic acid, glycyrrhizic acid and 4ʹ-o-glucosyl-5-methylvisamminol, were used for quality control. The concentration of each chemical marker was as follows: ferulic acid was 0.24 mg/g EK-6, 4ʹ-o-glucosyl-5-methylvisamminol was 0.54 mg/g, and glycyrrhizic acid was 3.45 mg/g.

### JHT treatment inhibited UVB-induced water loss from the skin and increased the TEWL

**Fig. 2:**
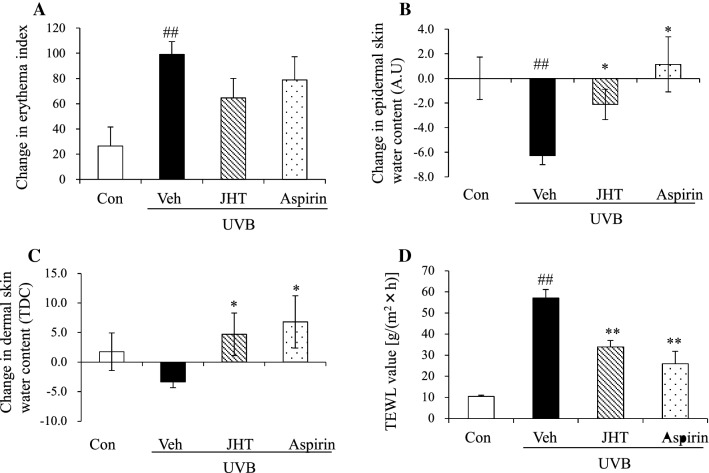
JHT treatment ameliorated UVB-induced skin damage. Indexes of UVB-induced skin damage were measured under isoflurane anesthesia at 72 h after irradiation. UVB-induced change in erythema index was not significantly affected by JHT treatment compared with the vehicle treatment (**a**). However, alterations in the content of skin water in the epidermis (**b**) and dermis (**c**) were alleviated in the JHT-treatment group relative to that in the vehicle group, and the value of transepidermal water loss (TEWL) was also improved by JHT treatment compared with that in the vehicle treatment (**d**). Data are expressed as means ± SEM (*n* = 4–5). ^##^*p* < 0.01 vs. control group; *p < 0.05, **p < 0.01 vs. vehicle-treatment group; Tukey test. *Con* control, *Veh* vehicle, *JHT* *Jumihaidokuto*, *A.U* Arbitrary unit, *TDC* Tissue dialectic constant

We first evaluated whether JHT treatment inhibited UVB-induced skin damage in hairless mice. In preliminary tests, we assessed the optimal time point for evaluating the effect of JHT, which revealed that at 72 h after UVB irradiation, TEWL and erythema were markedly increased and epidermal water content was decreased (data not shown). Therefore, in this study, we focused on the skin parameters at 72 h post-irradiation. We treated mice with vehicle or JHT (1000 mg/kg, p.o.) or aspirin (30 mg/kg, p.o.)—a COX-2 inhibitor—which was used as a positive control, for 3 weeks before UVB irradiation, and measured the skin parameters before and after UVB irradiation (Fig. 2). A single dose of UVB irradiation significantly increased the erythema index and reduced the skin water content in the epidermis, and, compared with the vehicle treatment, pretreatment with JHT or aspirin significantly inhibited the reduction in skin water content in the epidermis but not the erythema index (Fig. 2a–b). UVB irradiation also tended to reduce the skin water content in the dermis, although this reduction was not statistically significant, and pretreatment with JHT or aspirin significantly increased the skin water content in the dermis compared with the vehicle treatment (Fig. 2c). Furthermore, we measured the TEWL values after irradiation to evaluate the effect of JHT on the skin barrier function. UVB irradiation increased TEWL significantly relative to the level in the control group, and pretreatment with JHT or aspirin inhibited the increase in TEWL (Fig. 2d).

### JHT treatment inhibited UVB-induced subcutaneous swelling and epidermal hyperplasia

**Fig. 3:**
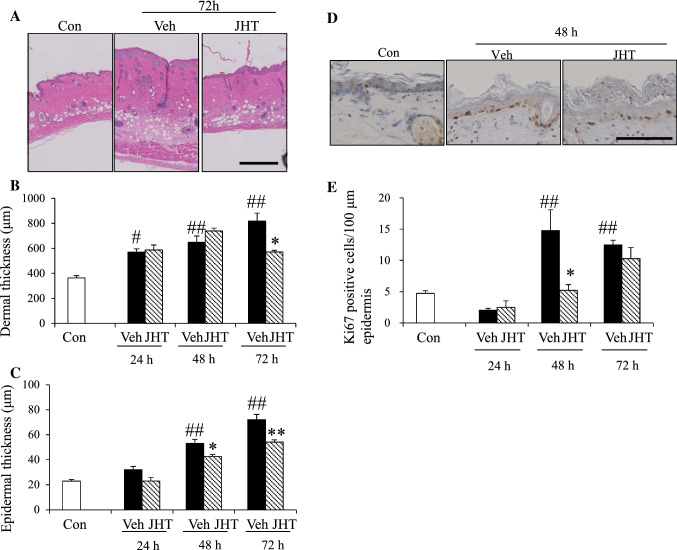
JHT treatment ameliorated UVB-induced subcutaneous swelling and epidermal hyperplasia. Dorsal skin tissue was stained with hematoxylin and eosin (HE) (**a**) and the thickness of dermis and epidermis was evaluated. JHT treatment inhibited the increase in dermal thickness only at 72 h after irradiation (**b**) but suppressed the increase in epidermal thickness at 48–72 h after irradiation (**c**). Data are expressed as means ± SEM (*n* = 4–5). Scale bar = 500 μm. Ki67-positive cells in the epidermis were examined after irradiation (**d**–**e**); quantification of the results revealed that after UVB irradiation, the number of Ki67-positive cells was decreased at 24 h and increased at 48 and 72 h post-irradiation. JHT treatment inhibited the increase at 48 h after irradiation (**e**). Data are expressed as means ± SEM (*n* = 4–5). ^#^*p* < 0.05, ^##^*p* < 0.01 vs. control group; Turkey test. *p < 0.05, **p < 0.01 vs. vehicle-treatment group; Student’s *t* test. Scale bar = 100 μm. *Con* control, *Veh* vehicle, *JHT* *Jumihaidokuto*

To evaluate the effect of JHT on swelling and epidermal hyperplasia after UVB irradiation, we stained the samples with HE and measured skin thickness in the dermis and epidermis (Fig. 3a). UVB irradiation increased skin thickness in the dermis in a time-dependent manner, and JHT pretreatment inhibited this thickening of the skin at 72 h, but not at 24 or 48 h, after irradiation (Fig. 3b). UVB irradiation also increased epidermal skin thickness in a time-dependent manner, and in this case, JHT suppressed the increase in thickness at 48 and 72 h post-irradiation (Fig. 3c). These results suggest that the JHT prevents UVB-induced swelling and epidermal hyperplasia. In addition to the effect of JHT on skin thickness, we evaluated the effect on the proliferation of keratinocytes by immunostaining for the proliferation marker, Ki67 (Fig. 3d). One of the mechanisms involved in UVB-induced epidermal hyperplasia is reported to be the dysregulation of keratinocyte proliferation [22]. Following UVB irradiation, the number of Ki67-positive cells was decreased at 24 h and increased at 48 and 72 h, and JHT pretreatment reduced the UVB-induced increase in Ki67-positive cells at 48 h post-irradiation but produced no change (relative to the vehicle treatment) at 24 or 72 h (Fig. 3e).

### JHT treatment inhibited UVB-induced degradation of the ECM

**Fig. 4:**
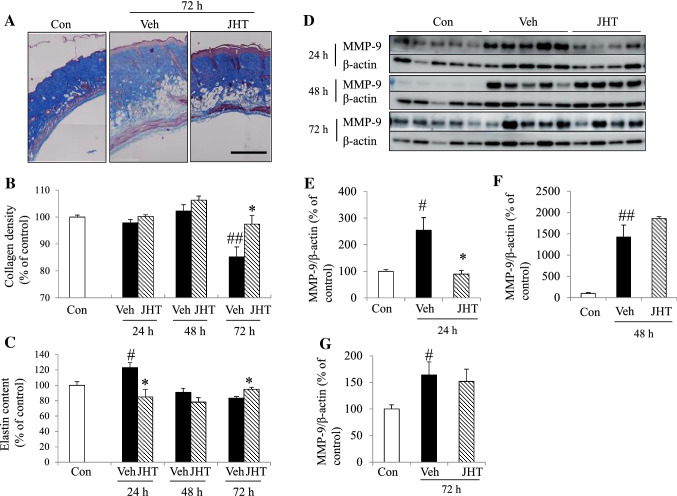
JHT treatment inhibited UVB-induced degradation of extracellular matrix (ECM) and expression of MMP-9. Collagen content in the dermis was evaluated through Masson’s trichrome staining (**a**), and elastin content in the skin was measured using ELISA (**c**). UVB irradiation decreased the collagen content, and JHT treatment inhibited the collagen-content reduction at 72 h post-irradiation (**b**). UVB increased the elastin content at 24 h after irradiation, and the content then decreased at 72 h post-irradiation; JHT treatment inhibited the effect of UVB on elastin content (**c**). Data are expressed as means ± SEM (*n* = 4–5). ^#^*p* < 0.05, ^##^*p* < 0.01 vs. control group; Tukey test. *p < 0.05 vs. vehicle-treatment group; Student’s *t* test. To evaluate the expression of MMP-9 after irradiation, western blot analysis was performed (**d**–**g**). Quantification of the results revealed that after UVB irradiation, the expression level of MMP-9 was increased at all time points examined, and that JHT treatment inhibited the increase in expression at 24 h post-irradiation. Data are expressed as means ± SEM (*n* = 4–5). ^#^*p* < 0.05, ^##^*p* < 0.01 vs. control group; *p < 0.05 vs. vehicle-treatment group; Tukey test. *Con* control, *Veh* vehicle, *JHT* *Jumihaidokuto*

We next examined how collagen and elastin contents in the dermis were affected by JHT. To assess the collagen content, we performed Masson’s trichrome staining (Fig. 4a). UVB irradiation significantly reduced the collagen content in the dermis only at 72 h relative to the control level, and this reduction at 72 h post-irradiation was significantly inhibited by pretreatment with JHT (Fig. 4b). To evaluate the content of elastin in the skin, we performed ELISA. The single-dose UVB irradiation significantly increased the elastin content at 24 h and decreased the content at 72 h compared with the level in the control group, and pretreatment with JHT significantly inhibited this increase and decrease in elastin content at 24 and 72 h, respectively (Fig. 4c). To investigate how JHT ameliorated the reduction in collagen content, we examined the expression of MMP-9 in skin (Fig. 4d–g). UVB irradiation significantly increased the expression levels of MMP-9 at 24–72 h, and pretreatment with JHT significantly inhibited this upregulation of MMP-9 only at 24 h post-irradiation (Fig. 4e).

### JHT treatment did not inhibit UVB-induced oxidative stress on DNA or reduction in the expression of antioxidant enzymes

**Fig. 5:**
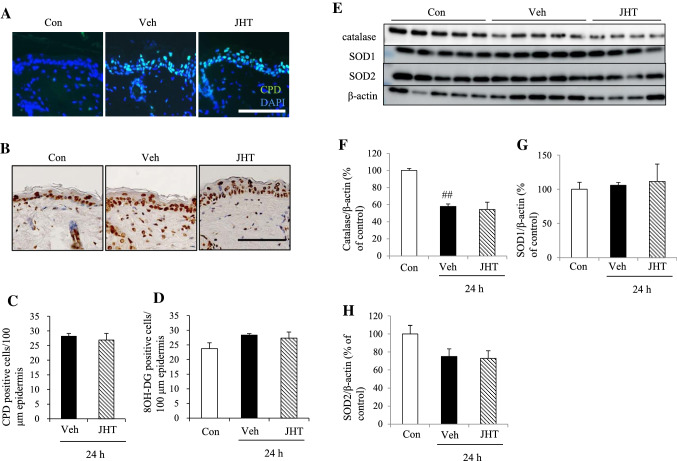
JHT treatment did not inhibit UVB-induced DNA damage or downregulation of antioxidant enzymes. To evaluate UVB-induced DNA damage, CPD- and 8OH-DG-positive cells in the epidermis were identified and quantified through immunohistochemical staining at 24 h post-irradiation. No CPD-positive cells were detected in the control group, and after UVB irradiation, the number of CPD-positive cells was increased; this number did not differ between the JHT-treatment and vehicle-treatment groups (**a**, **c**). UVB irradiation slightly increased the number of 8OH-DG-positive cells, but this number also did not differ between the JHT-treatment and vehicle-treatment groups (**b**, **d**). Scale bar = 100 μm. Western blotting was used to evaluate the expression levels of the antioxidant enzymes catalase, SOD1, and SOD2 at 24 h post-irradiation (**e**). UVB irradiation decreased the expression of catalase, but JHT treatment did not affect this change (**f**), and neither the irradiation nor the JHT treatment altered the expression of SOD1 (**g**) or SOD2 (**h**). Data are expressed as means ± SEM (*n* = 4–5). ^##^*p* < 0.01 vs. control group; Tukey test. *Con* control, *Veh* vehicle, *JHT* *Jumihaidokuto*

To investigate the mechanism of action of JHT, we evaluated the antioxidant activity of JHT in hairless mice. Oxidative stress is widely recognized to play a pivotal role in initiating the signaling pathways that are involved in the degradation of ECM [1], and JHT was previously reported to produce an antioxidant effect in an in vitro assay [19]. First, we measured the number of CPD-positive cells in the epidermis to assess the effect of JHT on direct DNA damage at 24 h after UVB irradiation. In the control group, no CPD-positive cells were detected in the epidermis, and UVB irradiation increased the number of CPD-positive cells significantly, but this increased number was not altered by pretreatment with JHT (Fig. 5a, c). Second, we quantified the 8OH-DG-positive cells in the epidermis to examine the effect of JHT on indirect DNA damage through ROS production. In the control group, almost all cells in the epidermis were 8OH-DG-positive, and no difference was measured among the groups (Fig. 5b, d). Third, we evaluated the expression levels of the antioxidant enzymes, catalase, SOD1, and SOD2 (Fig. 5e–h): UVB irradiation reduced the expression of catalase at 24 h post-irradiation and pretreatment with JHT did not affect this reduction in the expression (Fig. 5f), and neither did UVB irradiation or JHT treatment exert any effect on the expression levels of SOD1 or SOD2 at 24 h post-irradiation (Fig. 5g–h).

### JHT treatment inhibited UVB-induced infiltration of neutrophils and macrophages in the dermis

**Fig. 6:**
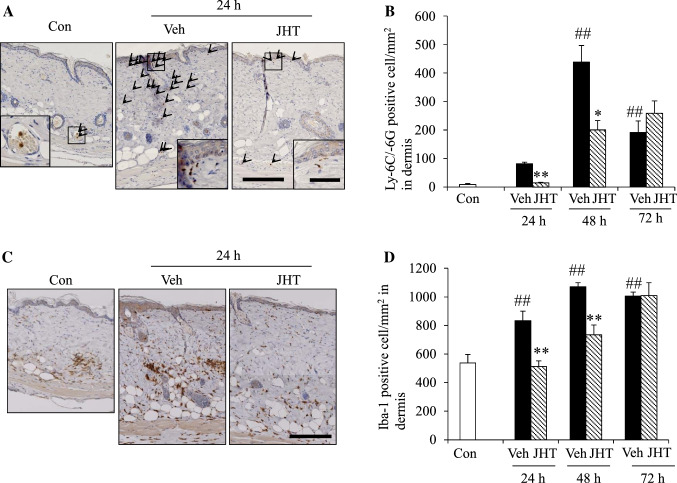
Pretreatment with JHT inhibited UVB-induced infiltration of immune cells. Immunohistochemical (IHC) staining was used to evaluate neutrophils in the dermis. Neutrophils were identified and quantified as Ly-6G/-6C-positive cells (**a**; arrowheads). UVB irradiation increased the infiltration of neutrophils in the dermis in a time-dependent manner, and JHT treatment inhibited the infiltration at 24 and 48 h post-irradiation (**b**). Macrophages in the dermis were identified and quantified as Iba-1-positive cells through IHC staining (**c**). UVB irradiation increased the infiltration of macrophages in the dermis in a time-dependent manner, and JHT treatment inhibited the infiltration at 24 and 48 h after irradiation (**d**). Scale bar = 200 μm (low magnification) or 50 μm (high magnification). Data are expressed as means ± SEM (*n* = 4–5). ^#^*p* < 0.05, ^##^*p* < 0.01 vs. control; Turkey test. *p < 0.05, **p < 0.01 vs. vehicle-treatment group; Student’s *t* test. *Con* control, *Veh* vehicle, *JHT* *Jumihaidokuto*

Next, we quantified the neutrophils and macrophages that infiltrated the dermis because JHT was reported to modify the functions of both these cells [17, 18]. To evaluate neutrophil infiltration in the skin, we immunostained for the neutrophil marker, Ly-6C/-6G (Fig. 6a). In the control group, neutrophils were mainly detected within blood vessels and were rarely present in the dermis, whereas in the UVB irradiation group, neutrophils were abundant in the dermis and the cell numbers peaked at 48 h post-irradiation; notably, pretreatment with JHT significantly inhibited neutrophil infiltration at 24 and 48 h post-irradiation compared with that in the vehicle treatment (Fig. 6b). Next, we immunostained for Iba-1 to evaluate the effect on macrophage infiltration (Fig. 6c). UVB irradiation also increased the number of Iba-1-positive macrophages in a time-dependent manner, and pretreatment with JHT inhibited the macrophage infiltration at 24 and 48 h but not at 72 h post-irradiation (Fig. 6d).

### JHT treatment inhibited UVB-induced levels of proinflammatory mediators but not of COX-2

**Fig. 7:**
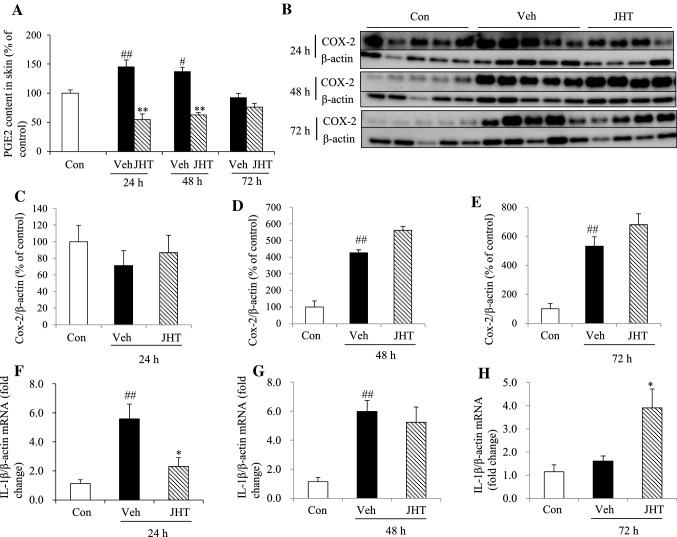
JHT treatment inhibited UVB-induced production of PGE2 and IL-1β. PGE2 content in the skin was measured using ELISA (**a**). UVB irradiation increased the content of PGE2 in the skin at 24 and 48 h, and JHT treatment reduced the content relative to that in the vehicle treatment. ^#^*p* < 0.05, ^##^*p* < 0.01 vs. control group; Tukey test. **p < 0.01 vs. vehicle-treatment group; Student’s *t* test. COX-2 expression in the skin was evaluated through western blotting (**b**). Quantification of the results revealed that after UVB irradiation, the expression of COX-2 was increased at 48 (**d**) and 72 h (**e**) but not at 24 h (**c**), and JHT treatment did not alter the expression of COX-2 at any time point. Data are expressed as means ± SEM (*n* = 4–5). ^##^*p* < 0.01 vs. control group; Tukey test. Real-time PCR was used to evaluate the levels of IL-1β mRNA (**f**–**h**). After UVB irradiation, IL-1β mRNA was increased at 24 (**f**) and 48 h (**g**) and then decreased back to the control level at 72 h (**h**). JHT treatment inhibited the increase in the levels of IL-1β mRNA at 24 h, and, at 72 h, caused an increase in the mRNA level relative to the level in the vehicle-treatment group. Data are expressed as means ± SEM (*n* = 4). ^##^*p* < 0.01 vs. control group, *p < 0.05 vs. vehicle-treatment group; Tukey test. *Con* control, *Veh* vehicle, *JHT* *Jumihaidokuto*

UVB-induced erythema is associated with increased vascular permeability mediated by PGE2, which is widely recognized as a key signaling molecule in UVB-triggered inflammation. Accordingly, after UVB irradiation, the expression of PGE2 was increased at 24 and 48 h, but not at 72 h, compared with the level in the control group, and JHT treatment significantly inhibited the upregulation of PGE2 at 24 and 48 h post-irradiation (Fig. 7a). In the UVB-induced inflammation model, PGE2 synthesis from arachidonic acid is mainly regulated by the enzyme COX-2, and therefore, we investigated the effect of JHT on the expression of COX-2 by western blot analysis (Fig. 7b). UVB irradiation significantly increased the expression of COX-2 at 48 and 72 h post-irradiation, but JHT treatment did not inhibit this elevation in the expression of COX-2 relative to that in the vehicle treatment at any time point post-irradiation (Fig. 7c–e). Lastly, we investigated whether JHT affected the expression of IL-1β. After UVB exposure, the levels of IL-1β mRNA were increased at 24 and 48 h, but not at 72 h, post-irradiation compared with the level in the control group, and JHT treatment lowered and elevated the IL-1β levels at 24 and 72 h post-irradiation, respectively, relative to the respective levels in the vehicle-treatment group (Fig. 7f–h).

## Discussion

We investigated whether and how oral JHT treatment affects UVB-triggered skin sunburn using the UVB-induced skin damage model in HR-1 hairless mice. JHT administration prevented UVB-induced skin damage, such as loss in skin moisture and increase in TEWL, but not erythema, and JHT also inhibited UVB-induced degradation of ECM and infiltration of immune cells. Furthermore, JHT treatment partly inhibited UVB-induced production of PGE2 and proinflammatory cytokines. This is the first report to reveal how JHT treatment reduces UVB-triggered inflammatory damage of the skin.

Oxidative stress is considered to play a central role in initiating and driving the signaling pathway that leads to the cellular response following UVB irradiation [1]. Since one method of ameliorating UVB-induced skin damage involves the use of antioxidants for radical scavenging and ROS quenching, numerous natural products, such as plant extracts and herbs, have been developed as sunscreen materials for their antioxidant activity [2, 11–13]. However, we found here that oral administration of JHT did not decrease the number of 8OH-DG- and CPD-positive cells in the epidermis at 24 h post-irradiation (Fig. 5), and JHT treatment also did not inhibit the change in the expression of antioxidant enzymes in the skin (Fig. 5). Although JHT was previously reported to inhibit neutrophil-derived and cell-free ROS induction in an in vitro assay [19], our results suggest that JHT exerts no radical-scavenging or ROS-diminishing effect through antioxidant enzymes in this model.

UVB irradiation enhances the production of proinflammatory mediators from various skin cells through the induction of ROS production and the infiltration of immune cells, such as neutrophils and macrophages, into the skin. One of the key signaling molecules in UVB-induced inflammation is PGE2. PGE2 is also widely recognized to increase vascular permeability and, thereby, facilitates the infiltration of neutrophils and macrophages from the bloodstream, which leads to swelling and edema at the site of inflammation. Furthermore, proinflammatory cytokines, such as IL-1β, also play a pivotal role in the acute phase of inflammation and enhance the expression of adhesion molecules, such as VCAM-1 on endothelial cells; this enables leukocytes to adhere to the wall of vessels for the infiltration of immune cells [23]. We show that JHT treatment decreased the expression of both PGE2 and IL-1β (Fig. 7a, f) and inhibited the infiltration of immune cells into the dermis (Fig. 6). Thus, downregulation of PGE2 or IL-1β might contribute to the inhibition of the infiltration of immune cells.

During PGE2 synthesis, membrane-released arachidonic acid is rapidly oxidized into an unstable metabolite, PGG2, which is then reduced to PGH2; COX enzymes sequentially catalyze both of these steps. COX-2 is widely recognized to mediate UVB-induced production of PG in the skin. Following COX-2-catalyzed synthesis of PGH2, three distinct synthases convert PGH2 to PGE2: microsomal PGE synthase-1 (mPGES-1), mPGES-2, and cytosolic PGE synthase. Increased expression of COX-2 and PGE2 has been directly associated with several UVB-induced skin inflammatory reactions, including swelling, erythema, epidermal hyperplasia, and keratinocyte proliferation [24, 25]. Here, we demonstrated that JHT treatment inhibited UVB induction of swelling, proliferation of keratinocytes, and PGE2 content, but did not inhibit the expression of COX-2 (Figs. 3, 7a–e). These results indicate that JHT might act as a direct or indirect inhibitor of COX-2 or another PGE2 synthase. Intriguingly, other COX-2 inhibitors, such as celecoxib, are reported to inhibit UVB-induced skin carcinogenesis [26]. In this study, aspirin treatment produced a similar effect as JHT treatment on the content of skin water and TEWL (Fig. 2).

PGE2-stimulated increase in vascular permeability is also associated with erythema induced by UVB. However, in this study, treatment with JHT or aspirin did not inhibit erythema at 72 h post-irradiation compared with the vehicle treatment (Fig. 2a). Notably, the expression of PGE2 was increased at 24 and 48 h post-irradiation, but not at 72 h post-irradiation, when the erythema index was increased (Fig. 7a). These results suggest that PGE2 might not play any role in the induction of erythema at 72 h. Here, we assessed the erythema index by obtaining measurements at the specific wavelength that corresponds to the spectral absorption peak of hemoglobins. Subcutaneous hemoglobin content is considered to be affected by the structural and functional changes induced in blood vessels and lymphatic vessels by UVB [27]. Thus, dysfunction of the cutaneous vascular system might affect the induction of erythema more critically than it affects the PGE2 function at 72 h post-irradiation. Although we did not investigate this, JHT might not exert any effect on the cutaneous vascular system.

Photoaged skin is characterized by the UV-induced damage of ECM integrity that is responsible for the formation of wrinkles [28]. Within the ECM, the major structural proteins are collagen and elastin, and proteolytic enzymes, such as MMPs and elastases, which are produced by epidermal keratinocytes and fibroblasts, mediate the remodeling of ECM. UVB irradiation has been reported to alter the signal transduction pathways that promote the expression of MMP and elastase and decrease the synthesis of procollagen [29, 30]. MMP-9, which is a gelatinase, degrades both collagen fragments generated by other collagenases and elastic fibers [28], and IL-1β, besides affecting endothelial cells, is reported to increase the expression of MMP-9 in murine macrophages [31]. Here, JHT treatment inhibited the expression of both MMP-9 and IL-1β at the same time point after irradiation (Figs. 4d–g, 7 f–h). Although reduction in the content of collagen was observed at 72 h post-irradiation, inhibition of the expression of MMP-9 by JHT might partly contribute to the prevention of UVB-induced degradation of ECM. Similarly, in relation to elastin, a correlation is considered to exist between the content of elastin and formation of wrinkles in elderly people [32]. In photoaged skin, dystrophic elastotic material is accumulated in the dermis, which is referred to as solar elastosis [33, 34]. Accumulation of elastin fibers is considered to occur due to their overproduction and decreased degradation. Accordingly, UVB irradiation was shown to activate the elastin promoter and thus lead to increased elastin production and accumulation of abnormal elastin fibers in photoaged skin [7]. Since both macrophage-secreted MMP-12 and neutrophil-produced elastase are involved in degrading the elastin fibers, immune-cell infiltration enhances the degradation of ECM after UVB irradiation. By contrast, UVA and IL-1β are reported to enhance the expression level of elafin, a protein that inhibits neutrophil elastase-induced degradation of elastin [35]. In this study, after UVB irradiation, the elastin content was increased at 24 h and decreased at 72 h compared with the content in the control group (Fig. 4c). Furthermore, UVB also increased the level of IL-1β mRNA at 24 h post-irradiation, and this was decreased to the same level as in the control group at 72 h after irradiation (Fig. 7f–h). These results suggest that UVB could increase the production of elastin and decrease the degradation of elastin fibers through elafin activity at 24 h post-irradiation, and that MMP-12 and neutrophil elastase might disassemble the elastin fibers at 72 h post-irradiation. Conversely, JHT treatment decreased the expression of IL-1β mRNA at 24 h and increased the expression at 72 h post-irradiation compared with the vehicle treatment (Fig. 7f–h). Thus, JHT might inhibit solar elastosis and degradation of elastin through the modification of elafin activity. However, further investigation is necessary to clarify the detailed mechanism of action of JHT in skin inflammation.

Certain limitations of this study are as follows: first, we stopped administering JHT after UVB irradiation to avoid influencing skin inflammation by holding the animals. Moreover, in a few cases, we did not detect any differences between the vehicle-treatment and JHT-treatment groups at 72 h post-irradiation (Figs. 3d, 6, 7), and in terms of the expression of IL-1β mRNA, the level was higher in the JHT-treatment group than in the vehicle-treatment group at 72 h post-irradiation (Fig. 7h). This could have occurred because of all the active compounds contained in JHT having been metabolized by the 72-h time point. Intriguingly, cherry bark extract, a crude drug contained in JHT, was reported to promote the inflammatory response and accelerate wound healing in a mouse model of atopic dermatitis [36], which suggests that JHT can potentially modify the inflammatory response peak and thus facilitate the treatment of atopic dermatitis. Therefore, in this study, JHT treatment might have only delayed the inflammatory response peak after UVB irradiation and not completely prevented UVB-induced skin damage. Second, we did not investigate how many times JHT treatment is needed to prevent UVB-induced damage. In this study, mice were treated with JHT for 3 weeks before UVB irradiation. However, in other studies, mice were treated for less than 1 week to prevent inflammation [37, 38]. Thus, JHT treatment for shorter time might also prevent UVB-induced skin damage.

In conclusion, this study demonstrates that oral JHT treatment prevents UVB-induced loss in skin water, increase in TEWL, degradation of ECM, and infiltration of immune cells, and further that the protective effect of JHT against UVB-induced skin damage might be mediated by the downregulation of the expression of PGE2 and IL-1β. Moreover, these results suggest that oral administration of JHT could represent a favorable strategy for preventing UVB-induced skin sunburn.
